# Increases in Cognitive Activity Reduce Aging-Related Declines in Executive Functioning

**DOI:** 10.3389/fpsyt.2021.708974

**Published:** 2021-07-29

**Authors:** Mirjam Stieger, Margie E. Lachman

**Affiliations:** Lifespan Developmental Psychology Laboratory, Department of Psychology, Brandeis University, Waltham, MA, United States

**Keywords:** cognition, cognitive engagement, educational attainment, executive function, episodic memory, cognitive aging

## Abstract

Declines in cognitive functioning are commonly experienced with aging, yet there is wide variation in the nature and extent of these changes. Previous research has shown associations between the frequency of engaging in stimulating cognitive activities and performance on cognitive tests. However, it is not known whether it is important to increase the amount of cognitive activity as one gets older in order to curtail cognitive declines. We examined cognitive activities and cognition in a national sample of 2,130 community-dwelling middle-aged and older adults from the Midlife in the United States longitudinal study. Participants completed cognitive assessments of episodic memory (EM) and executive functioning (EF) on two occasions, ~9 years apart. At the first assessment, participants ranged in age between 33 and 83 years (*M* = 55.76, *SD* = 11.09), with 56.1% women. Multilevel models were tested to examine educational level and change in cognitive activity as predictors of change in EM and EF, controlling for initial level of cognitive activity and key correlates of cognitive decline. Results indicated that increases in cognitive activity were important over and above earlier levels of cognitive activity for reducing declines in EF. Further analysis also showed differential results by educational level. Increased cognitive activity was not associated with changes in EF among individuals with a college degree. In contrast, individuals without a college degree who increased their cognitive activity showed significantly less decline in EF compared to those without a college degree who decreased or maintained their cognitive activity across the 9 years. Thus, the results suggest that increased engagement in cognitive activity makes more of a difference for EF declines among those without a college degree. The results have particular relevance given that aging-related changes in domains such as work status, health, or social relationships can lead to reductions in stimulating cognitive activities. The findings emphasize the importance of increasing cognitive activities especially among those with lower educational attainment.

## Introduction

There are wide individual differences in cognitive declines with aging (1–4), with variations in the extent and timing. For some individuals, declines occur earlier or faster and others are better able to maintain their cognitive functioning as they age (1, 3, 5, 6). Maintenance of cognitive functioning into old age is desirable for the individual as well as for society (7) as it promises enhanced quality of life (8), prolongs the time span in which individuals can live independently, and reduces the overall costs of long-term care (9).

There is much interest in identifying modifiable behaviors and lifestyle factors that can reduce cognitive impairment and dementia. Engagement in cognitive activities such as reading books or playing word games is a modifiable lifestyle factor that can potentially mitigate cognitive declines. Indeed, prior research found that participation in everyday cognitive activities is related to better cognitive functioning (10), a reduced rate of cognitive decline (11), and a lower risk of developing mild cognitive impairment, dementia (12), or Alzheimer's disease (11, 13). However, an individual's engagement in cognitive activities may change in midlife and old age due to changes in work status, social engagement, or health. Thus far, it is unclear if changes in the frequency of cognitive activities is linked to the rate of cognitive decline and, more specifically, if increased engagement in cognitive activities can attenuate the rate of cognitive decline.

Educational attainment is seen as another protective factor that is related to cognitive functioning (14) but the link between educational attainment and age-related declines in cognitive performance is less clear. There exist at least three theoretical explanations on how educational attainment might be linked to cognitive declines. First, individuals with higher educational attainment may show attenuated rates of cognitive declines because they might have an increased capacity to counteract negative brain changes in aging for example through compensatory strategies (15). Second, individuals with higher educational attainment may be able to delay the onset of cognitive decline due to auxiliary brain structures. However, they might show faster cognitive declines once these auxiliary brain structures begin to deteriorate (16). Third, educational attainment may alter one's level of cognitive function but not attenuate one's rate of cognitive decline (15, 17). In recent years, there has been a growing debate on the direct impact of educational attainment on the timing and extent of cognitive declines. Many empirical studies have found an association between more years of education and later onset and attenuated rates of cognitive declines (18), and a lower risk of developing dementia or Alzheimer's disease (19), although others have not found this association (20, 21). These inconsistencies in prior studies emphasize that other factors such as one's engagement in cognitive activities in midlife and old age may influence the relationship between education and cognitive change. In general, individuals with higher educational attainment are inclined to engage more in stimulating cognitive activities, which may enhance their cognitive reserve (22). Of interest is whether middle-aged and older adults with lower educational attainment who increase their engagement in cognitive activities in later life can reduce the rate of cognitive decline.

Past cross-sectional research found that individuals without a college degree who frequently engage in cognitive activities showed similar scores in EM to individuals with a college degree (22). This highlights the potentially beneficial effects of cognitive activities to compensate for negative effects of aging-related factors that are less modifiable in later life such as educational attainment. Although the findings of this prior cross-sectional study suggest that more frequent engagement in cognitive activity is associated with better cognitive performance among individuals without a college degree (22), it is not clear if *changes* in the frequency of engaging in cognitive activities, which may occur due to retirement (23) and other aging-related experiences, are related to *changes* in cognition, and whether this varies by level of educational attainment.

For the current research, we used data from the national longitudinal observational study Midlife in the United States (MIDUS) with two assessments 9 years apart. The goal of this study was to examine if changes in the frequency of engagement in cognitive activities are related to the magnitude of cognitive decline in EM and EF over 9 years in middle-aged and older adults, and whether the effects varied by educational attainment. The specific research goals were two-fold. The first goal was to examine if change in cognitively stimulating activity is associated with change in cognitive functioning over 9 years. It was expected that individuals who increased their cognitive activity would show reduced declines in cognitive performance. The second goal was to examine if the effects of cognitive activity apply equally for individuals with or without a college degree. Increased engagement in cognitive activities was expected to attenuate aging-related cognitive declines especially among those with less education.

## Methods and Materials

### Study Sample

Participants of the present study were from two measurement waves of the Midlife in the United States study (MIDUS) in 2004–2006 (M2) and 2013–2014 (M3). A total of 2,130 participants completed the cognitive assessment at both occasions and were thus included in this study. The sample ranged in age between 33 and 83 years (*M* = 55.76, *SD* = 11.09) at the first occasion and women compromised 56.1% of the sample. On average, the cognitive tests at M2 and M3 were conducted 9.32 years apart (*SD* = 0.45). Descriptive statistics are shown in Supplementary Table 1. The Institutional Review Boards at the University of Wisconsin and Brandeis University approved the study. Details of the MIDUS longitudinal design, sampling, and all assessment instruments are available on the MIDUS website (http://midus.wisc.edu/). Also, the MIDUS data is publicly available at https://midus.colectica.org/ or https://www.icpsr.umich.edu/web/ICPSR/search/studies?q=midus.

Initially, the MIDUS study included 7,108 non-institutionalized participants in 48 states selected via random digit phone dialing which were assessed in 1995–1996 (M1), stratified by age with an oversample of those between 40 and 60 years of age (24). Participants of M1 ranged in age between 24 to 75 years (*M* = 46.40, *SD* = 13.00), had a mean education level of 13.21 years, and women made up 48.3% of the sample. M1 was not included in this study because cognitive performance was not administered. As is typically found, those who participated longitudinally at M2 and M3 were positively selected on a number of variables compared with those who dropped out of the study (25). Compared to the dropouts, longitudinal participants were more highly educated, *t*_(6757)_ = 12.48, *p* < 0.001; were more likely to be women (53.8 vs. 48.3%), χ2_(1)_ = 17.49, *p* < 0.001; and had higher self-rated health, *t*_(6759)_ = 10.42, *p* < 0.001 (*M* = 3.61 vs. 3.33). Dropouts were more likely to be non-white (16%) compared to the longitudinal participants (7% non-white), χ2_(1)_ = 112.22, *p* < 0.001. Also, at M2, longitudinal participants engaged in cognitive activities more frequently [*t*_(4012)_ = −6.56, *p* < 0.001] compared to the dropouts. In addition, at M3, participants who completed both cognitive assessments had higher scores in EF [*t*_(3353.33)_ = −13.80, *p* < 0.001] and EM [*t*_(3364.96)_ = −9.51, *p* < 0.001) compared to those who only completed the cognitive assessment at M2. For more details on the comparison of longitudinal participants and dropouts see Hughes et al. (4).

### Independent and Dependent Variables

#### Education

Participants were asked for their highest grade of school completed and degrees earned. Participants were categorized as those without a college degree (0) or with a college degree (1).

#### Cognitive Activities

At both occasions, participants indicated the frequency of engaging in four cognitive activities: reading books, magazines or newspapers; doing word games such as crossword, puzzles, or scrabble; attending educational lectures or courses; and writing (e.g., letters, journal entries, or stories). For example, participants were asked “How often do you.read books, magazines, or newspapers?” and responded on a 6-point scale (1 = never to 6 = daily). The items were averaged. Based on their change scores (M3–M2), participants were categorized as those who decreased or maintained their level of cognitive activity over time (0) and those who increased their level of cognitive activity over time (1).

#### Cognitive Performance

At both occasions, two cognitive factors were assessed using the Brief Test of Adult Cognition by Telephone (BTACT) (26, 27). The BTACT assesses key cognitive domains that are of theoretical significance for cognitive aging, and was designed for telephone administration with a wide range of ages and levels of educational attainment (26, 28). The BTACT battery includes a combination of existing and new subtests, and is a reliable, valid measure of cognition, despite its brief length [for more information on psychometric properties, see Lachman et al. (27)]. Seven cognitive tests are included in the BTACT (27). This included inductive reasoning (number series; completing a pattern in a series of five numbers), category verbal fluency (the number of words produced from the category of animals in 60 s), working memory span (backward digit span; the highest span achieved in repeating strings of digits in reverse order), processing speed (30 Second And Counting Task, or 30-SACT; the number of digits produced by counting backward from 100 in 30 s), and attention switching and inhibitory control [Stop and Go Switch Task, SGST; Tun and Lachman (28)] as well as two measures of episodic memory (immediate and delayed free recall of 15 words). For the SGST, reaction times were calculated with the mean of switch and non-switch trials median latencies on a task requiring alternating between the “normal” condition (i.e., respond “Go” to the stimulus “Green” and “Stop” to the stimulus “Red”) and the “reverse” condition (i.e., respond “Stop” to the stimulus “Green” and “Go” to the stimulus “Red”). Studies examining the psychometric properties of the BTACT yielded a good model fit with a two-factor solution consisting of executive functioning and episodic memory (4, 22, 27) and the two-factor structure was found to be invariant across the two occasions (4). Prior research also shows that psychosocial, behavioral, and biological factors are related to these two cognitive factors and changes therein [e.g., (29–33)]. The EF factor score includes the scores for working memory span, verbal fluency, inductive reasoning, processing, and attention switching and inhibitory control. The EM factor score includes the scores for immediate and delayed word list recall. Higher numbers indicate better EF and EM. The M3 scores were standardized using means and standard deviations from M2.

### Covariates

A number of risk factors for cognitive impairment, dementia, and Alzheimer's disease that were administered at M2 were included as covariates as they might potentially influence the rate of cognitive decline (13, 34). Thus, we could examine the independent effects of cognitive activity change over and above the included covariates. We also controlled for initial level of cognitive activity.

#### Demographic Variables

Age, sex (0 = women, 1 = men), and total household income in dollars were examined.

#### Functional Health

Ten questions were used to assess participant's functional health (35): How much does your health limit you in lifting or carrying groceries; bathing or dressing yourself; climbing several flights of stairs; climbing one flight of stairs; bending, kneeling, or stooping; walking more than a mile; walking several blocks; walking one block; vigorous activity (e.g., running, lifting heavy objects); moderate activity (e.g., bowling, vacuuming)? Responses were provided on a 4-point scale: 1 = a lot, 2 = some, 3 = a little, 4 = not at all. A mean score across the 10 items was computed with higher values representing better functional health.

#### Self-Rated Health

Participants rated their self-rated physical health on a 5-point scale: In general, would you say your physical health is 1 = poor to 5 = excellent?

#### Physical Activity

Twelve questions to assess vigorous and moderate intensity of physical activity were used. These questions referred to frequency of physical activities separately for the summer and winter months, in three different settings (i.e., home, work, and leisure), with ratings from 1 = never to 6 = several times a week. The mean across summer and winter in all three settings for both moderate and vigorous intensity was computed. The activity intensity and setting with the maximum value to represent the highest frequency of physical activity across all intensity levels and domains was selected (22).

#### Body Mass Index

Body mass index (BMI) was derived as kg/m^2^.

#### Depression

Depression was included as a dichotomized variable (0 = no, 1 = yes) based on seven questions about experiences with depressed affect and anhedonia over at least a two-week period in the previous 12 months. If an individual said yes to four or more statements about depressed affect or anhedonia and they stated that they felt it “everyday” or “almost every day,” the dichotomous variable was coded as “yes” to show that the individual met the criteria for depression.

#### Diabetes

Participants were asked if they have experienced or been treated for diabetes or high blood sure (0 = no, 1 = yes).

#### Head Trauma

Participants were asked if they have a history of a serious head injury (0 = no, 1 = yes).

#### Hypertension

Participants were asked if they have experienced or been treated for high blood pressure of hypertension (0 = no, 1 = yes).

#### Smoking

Participants were asked if they have ever smoked cigarettes regularly (0 = no, 1 = yes).

#### Sleep Problems

Participants were asked how often they have experienced trouble getting to sleep or staying asleep during the past 30 days (1 = almost every day to 6 = not at all).

#### Vitamin C

Participants were asked if they take vitamin C regularly (at least a couple of times a week) (0 = no, 1 = yes).

#### Alcohol or Drug Problems

Participants were asked if they have experienced or been treated for alcohol or drug problems in the past 12 months (0 = no, 1 = yes).

### Statistical Analysis

Longitudinal multilevel models in R (36) were used to investigate change in EM and EF over time. The data structure included repeated assessments of EM and EF (Level 1: Time) nested within participants (Level 2: Person). All continuous independent variables were grand mean centered.

To examine the association between education, change in cognitive activity and cognitive change, linear conditional models were fitted to test for differential effects over time. Mixed models use all available data and take into account that repeated measures on the same individual are correlated with each other. The models were estimated with ML. A time x cognitive activity change interaction term was added as a Level 2 predictor to investigate whether cognitive changes differed between those who decreased or maintained their level of cognitive activity over time and those who increased their level of cognitive activity over time. To test if the effect of cognitive activity change differed between individuals with and without a college degree, we added a time x cognitive activity change x education interaction term as a Level 2 predictor to the models. As a robustness check, these models were conducted with and without the covariates. We also controlled for initial level of cognitive activity in these models.

## Results

Table 1 reports descriptive statistics and stability correlations for the repeated measures. Supplementary Table 1 reports within occasion correlations among the study variables. Education was correlated with cognitive activity at both waves indicating that individuals with lower education engaged less frequently in cognitive activity. On average, participants had 14.58 years of education (*SD* = 2.67), which represents some years of college. Of the sample, 51.1% had a college degree and 48.8% did not have a college degree. A multilevel analysis suggests a significant overall change in cognitive activity over time (*B* = 0.04, *SE* = 0.02, 95% CI = −0.01; 0.08, *p* = 0.024). More specifically, 888 individuals increased (*M* = 0.78, *SD* = 0.53), 876 decreased (*M* = −0.68, *SD* = 0.51), and 366 maintained the same level of cognitive activities over time.

**Table 1:** Descriptive statistics.

		**M2**	**M3**	**Correlation M2 and M3**
	**Range**	***M (SD)*** **or %**	***M (SD)***	
Episodic memory (EM)	−2.10–3.64	0.11 (0.92)	−0.02 (0.99)	0.54
Executive functioning (EF)	−2.08–2.34	0.11 (0.64)	−0.15 (0.74)	0.77
Cognitive activity	1–6	3.09 (0.87)	3.13 (0.93)	0.59
Δ Cognitive activity (increase)		41.7%		
Education (no college degree)		48.9%		
Age (years)	33–92	55.76 (11.09)	64.86 (11.09)	
Self-rated health	1–5	3.71 (0.93)		
Functional health	1–4	3.44 (0.71)		
Income (dollars)	0–300000	75430.15 (60347.03)		
Physical activity frequency	1–6	4.70 (1.65)		
Sex (women)		56.1%		
BMI	15.60–82.31	27.83 (5.68)		
Depression (no)		90.3%		
Diabetes (no)		91.6%		
Head trauma (no)		96.8%		
Hypertension (no)		71.7%		
Smoking (no)		54.1%		
Sleep problems	1–6	3.98 (1.74)		
Vitamin C (no)		75.6%		
Alcohol or drug problems (no)		99.1%		

*N = 2,130*.

### Are Educational Attainment and Cognitive Activity Change Associated With Cognitive Changes?

**Figure 1:**
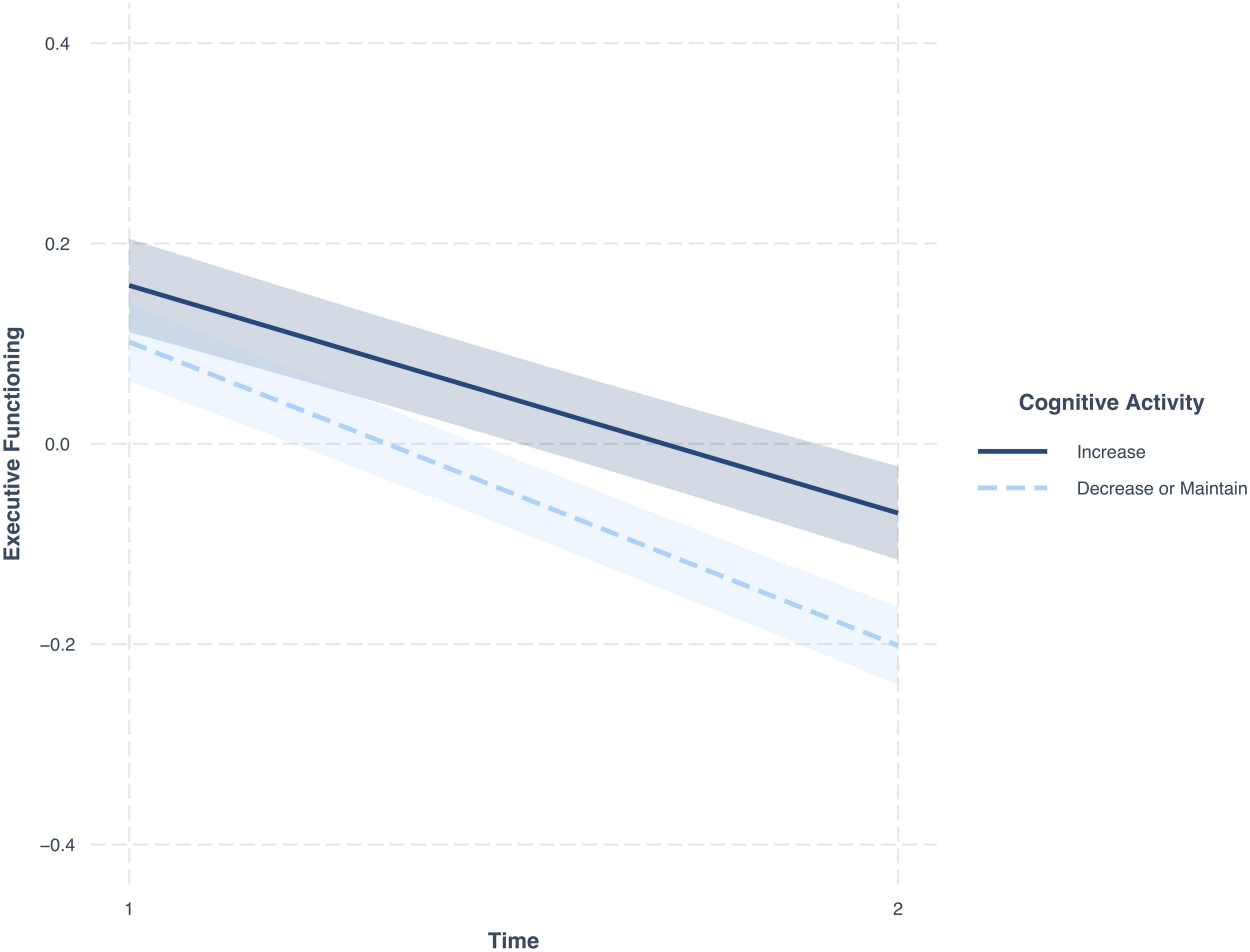
Changes in EF over time. Dark blue, change in individuals who increased their cognitive activity across the 9 years; Light blue, change in individuals who decreased or maintained their cognitive activity across the 9 years; Figure includes 95 percent confidence intervals; Covariates in model, cognitive activity at M2, income, age, sex, frequency of physical activity, functional health, self-rated health, BMI, depression, diabetes, head trauma, hypertension, smoking, sleep problems, vitamin C, alcohol or drug.

Table 2 shows the effect of education and change in cognitive activity on change in EF, controlling for the covariates and initial level of cognitive activity at M2. The results indicate that individuals with or without a college degree did not differ in their change in EF over time (Model 1). However, those who increased their cognitive activity over time showed less decline in EF compared to individuals who reduced or maintained their level of engagement in cognitive activities (Model 2) (*B* = 0.08, *SE* = 0.02, 95% CI = 0.03; 0.13, *p* = 0.002). See Figure 1. Supplementary Table 2 shows these models without covariates.

**Table 2:** Effects of change in cognitive activity and education on change in executive functioning (EF) with covariates^a^.

	**Model 1**				**Model 2**				**Model 3**			
	***B***	***SE***	***p***	**CI_**95**_**	***B***	***SE***	***p***	**CI_**95**_**	***B***	***SE***	***p***	**CI_**95**_**
Intercept	0.23	0.03	<0.001	0.17; 0.30	0.25	0.04	<0.001	0.18; 0.32	0.28	0.04	<0.001	0.19; 0.35
Time	−0.25	0.02	<0.001	−0.29; −0.22	−0.29	0.02	<0.001	−0.33; −0.25	−0.31	0.02	<0.001	−0.35; −0.26
Age	−0.24	0.02	<0.001	−0.27; −0.21	−0.25	0.02	<0.001	−0.28; −0.22	−0.25	0.02	<0.001	−0.27; −0.22
Sex	−0.07	0.01	<0.001	−0.10; −0.04	−0.08	0.01	<0.001	−0.10; −0.05	−0.07	0.01	<0.001	−0.10; −0.04
Income	0.07	0.02	<0.001	0.04; 0.10	0.07	0.02	<0.001	0.04; 0.10	0.07	0.02	<0.001	0.04; 0.10
Self-rated health	0.09	0.02	<0.001	0.05; 0.12	0.08	0.02	<0.001	0.05; 0.12	0.08	0.02	<0.001	0.05; 0.12
M2 cognitive activity	0.11	0.01	<0.001	0.09; 0.14	0.12	0.01	<0.001	0.10; 0.16	0.13	0.01	<0.001	0.10; 0.16
Education	0.32	0.05	<0.001	0.23; 0.41	0.32	0.05	<0.001	0.22; 0.41	0.26	0.06	<0.001	0.15; 0.38
Education × Time	−0.04	0.02	0.149	−0.08; 0.01	−0.04	0.02	0.145	−0.08; 0.01	0.01	0.03	0.759	−0.05; 0.07
Δ cognitive activity					−0.03	0.05	0.467	−0.13; 0.06	−0.10	0.07	0.142	−0.22; 0.03
Δ cognitive activity × Time					0.08	0.02	0.002	0.03; 0.13	0.13	0.04	<0.001	0.06; 0.20
Education × Δ cognitive activity × Time									−0.11	0.05	0.027	−0.21; −0.01
AIC	4074.65				4061.30				4059.85			
BIC	4200.03				4198.60				4209.11			
Log Likelihood	−2016.3				−2007.7				−2004.9			
*R^2^* Marginal	0.345				0.349				0.350			

*Δ Cognitive activity: 0, decreased and maintained cognitive activity over time; 1, increased cognitive activity over time; Education: 0, without a college degree; 1, with a college degree*;

a *Significant covariates are included in the table. Additional non-significant covariates are physical activity, functional health, BMI, depression, diabetes, head trauma, hypertension, smoking, sleep problems, vitamin C, alcohol or drug problems; Fit indices of null model: AIC, 7126.51; BIC, 7151.94; Log likelihood, −3559.3*.

Table 3 shows the effect of education and change in cognitive activity on change in EM, controlled for the covariates and initial level of cognitive activity at M2. The results indicate that education (Model 1) and change in cognitive activity (Model 2) were not related to change in EM. Supplementary Table 3 shows these models without covariates.

**Table 3:** Effects of change in cognitive activity and education on change in episodic memory (EM) with covariates.

	**Model 1**				**Model 2**				**Model 3^a^**			
	***B***	***SE***	***p***	**CI_**95**_**	***B***	***SE***	***p***	**CI_**95**_**	***B***	***SE***	***p***	**CI_**95**_**
Intercept	0.16	0.06	0.005	0.05; 0.27	0.10	0.07	0.138	−0.03; 0.23	0.12	0.07	0.103	−0.02; 0.27
Time	−0.15	0.03	<0.001	−0.22; −0.08	−0.12	0.04	0.002	−0.20; −0.05	−0.12	0.04	0.006	−0.21; −0.03
Age	−0.25	0.02	<0.001	−0.29; −0.21	−0.26	0.02	<0.001	−0.30; −0.22	−0.26	0.02	<0.001	−0.30; −0.22
Sex	0.24	0.02	<0.001	0.20; 0.27	0.24	0.02	<0.001	0.20; 0.27	0.24	0.02	<0.001	0.20; 0.27
Income	0.07	0.02	<0.001	0.03; 0.11	0.07	0.02	0.001	0.03; 0.11	0.07	0.02	0.001	0.03; 0.10
M2 cognitive activity	0.13	0.02	<0.001	0.10; 0.17	0.14	0.02	<0.001	0.10; 0.18	0.14	0.02	<0.001	0.10; 0.18
Education	0.14	0.08	0.083	−0.02; 0.30	0.14	0.08	0.091	−0.02; 0.30	0.09	0.11	0.382	−0.11; 0.30
Education × Time	−0.01	0.05	0.830	−0.10; 0.08	−0.01	0.05	0.834	−0.10; 0.08	−0.02	0.06	0.803	−0.14; 0.11
Δ cognitive activity					0.16	0.08	0.058	−0.01; 0.32	0.10	0.11	0.381	−0.12; 0.33
Δ cognitive activity × Time					−0.07	0.04	0.168	−0.16; 0.03	−0.07	0.07	0.282	−0.21; 0.06
Education × Δ cognitive activity × Time									0.01	0.10	0.890	−0.18; 0.20
AIC	6850.80				6850.80				6851.47			
BIC	6976.18				6988.10				7000.73			
Log likelihood	−3404.4				−3402.4				−3400.7			
*R^2^* marginal	0.214				0.215				0.216			

*Δ Cognitive activity: 0, decreased and maintained cognitive activity over time; 1, increased cognitive activity over time; Education: 0, without a college degree; 1, with a college degree*;

a *Significant covariates are included in the table. Additional non-significant covariates are physical activity, self-rated health, functional health, BMI, depression, diabetes, head trauma, hypertension, smoking, sleep problems, vitamin C, alcohol or drug problems; Fit indices of null model: AIC, 10943.9; BIC, 10969.34; Log likelihood, −5468.0*.

The correlations in Supplementary Table 1 show that, at both occasions, those who were more cognitively active had higher levels in EF and EM. We also tested the effects of M2 cognitive activity on change in cognitive activity (Supplementary Table 4), EF (Supplementary Table 5) and EM (Supplementary Table 6). These additional multilevel analyses show that participants with higher levels of cognitive activity at M2 decreased more in their cognitive activity, EF, and EM over time compared to those with lower levels of cognitive activity at the first occasion. Those who increased in cognitive activity from M2 to M3 had the lowest activity scores at M2 (*M* = 2.84, *SD* = 0.78) yet the highest activity scores at M3 (*M* = 3.62, *SD* = 0.84). We also tested if increased cognitive activity is associated with change in EF and EM over and above the association of baseline cognitive activity with EF and EM. Supplementary Table 7 shows that increased cognitive activity was associated with reduced declines in EF, over and above the effects of baseline cognitive activity on change in EF. Supplementary Table 8 shows that increased cognitive activity was not associated with change in EM.

### Do the Effects of Cognitive Activity Change Vary by Educational Attainment?

**Figure 2:**
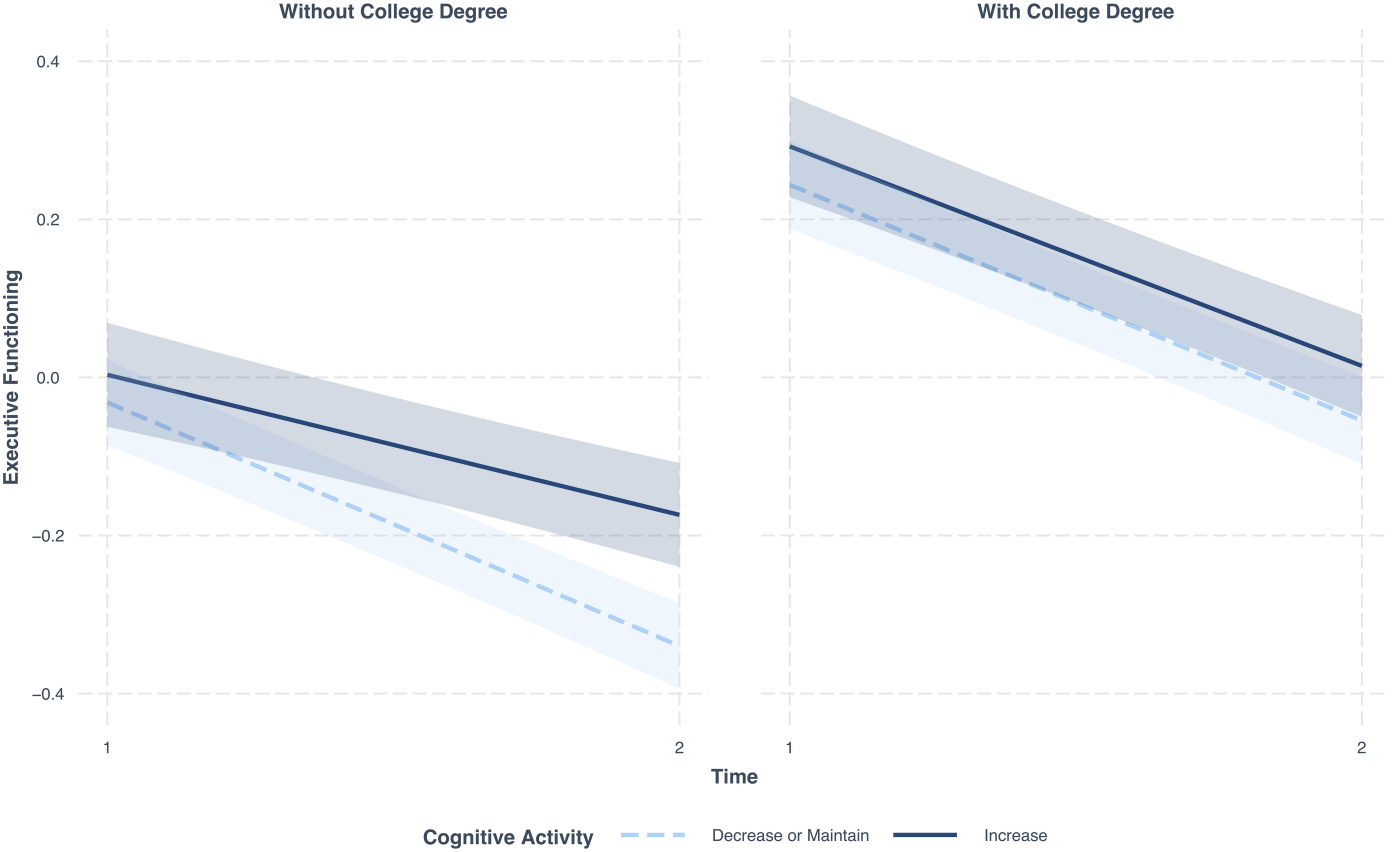
Changes in EF over time in individuals with a college degree (left) and without a college degree (right); Dark blue, change in EF in individuals who increased their cognitive activity across the 9 years; Light blue, change in EF in individuals who decreased or maintained their cognitive activity across the 9 years; Figures include 95 percent confidence intervals; Covariates in models, cognitive activity at M2, income, age, sex, frequency of physical activity, functional health, self-rated health, BMI, depression, diabetes, head trauma, hypertension, smoking, sleep problems, vitamin C, alcohol or drug.

Table 2 also shows that education moderated the effect of change in cognitive activity on change in EF (Model 3) (*B* = −0.11, *SE* = 0.05, 95% CI = −0.21; −0.01, *p* = 0.027). To specifically test these education differences, comparisons were examined for individuals with a college degree (*N* = 1,088; 51.1% of the sample) and without a college degree (*N* = 1040; 48.8% of the sample). The multilevel models show that among individuals with a college degree, the rate of cognitive change for individuals who increased their engagement in cognitive activity did not differ significantly (*p* = 0.554) from those who decreased or maintained their cognitive activity (Supplementary Table 9). However, individuals without a college degree who increased their cognitive activity over time showed significantly less decline in EF compared to individuals without a college degree who decreased or maintained their cognitive activity over time (*B* = 0.13, *SE* = 0.03, 95% CI = 0.07;0.20, *p* < 0.001) (Figure 2 and Supplementary Table 10).

Table 3 presents the moderation effects of education on change in EM (Model 3). Education did not moderate the effect of change in cognitive activity on change in EM.

## Discussion

The present study extends previous research by showing that those who increased in cognitive activity over a 9-year period showed less decline in EF than those who maintained or decreased in cognitive activity over and above the initial amount of cognitive activity. Moreover, further analysis showed that these effects were more pronounced for those with lower educational attainment. For the overall sample, results show that middle-aged and older adults who increased their cognitive activity showed reduced declines in EF. These findings contrast with those of a prior study with older adults ages 75–85, which found that higher level or maintenance of leisure activity was not protective against cognitive decline across a 10-year follow-up period (10). Participants who increased their cognitive activity from M2 to M3 in the present study had the lowest activity scores at M2 yet the highest activity scores at M3, which may help explain why increases in cognitive activity were advantageous in terms of mitigating cognitive declines. Our findings also suggest that participants with higher levels of cognitive activity at M2 decreased more in their cognitive activity, EF, and EM over time. These findings could be in part due to some regression to the mean, although it is typical to find that initial levels are negatively correlated with change, as the activity and cognitive measures do have some measurement error. The changes found in the cognitive measures [see Hughes et al. (4)], however, are consistent with theory and empirical studies of cognitive aging, in that both EF and EM decline over time in middle-aged and older samples, with individual differences in the magnitude of decline.

The present findings show a significant interaction of change in cognitive activity and education. Increased cognitive activity was not significantly associated with attenuated rates of EF declines among individuals with a college degree. In contrast, individuals without a college degree who increased their cognitive activity showed significantly less decline in EF compared to those without a college degree who decreased or maintained their cognitive activity across the 9 years. This could be because those without a college degree had lower initial levels of cognitive activity than their college educated counterparts, thus increases in cognitive activity would be more important for maintaining executive functioning. These findings are consistent with other studies that show that lifestyle factors or behavioral changes differentially help those who are more vulnerable to aging-related declines such as those with lower socioeconomic status, in this case those with less education (37). These effects were robust in that they held after controlling for baseline cognitive activity and a number of well-known risk factors for cognitive aging, dementia and Alzheimer's disease (13). The results highlight the importance of promoting and encouraging increased engagement in cognitive activity especially among those with less education, who typically are less cognitively active than their more educated counterparts. Moreover, adults are particularly vulnerable to cognitive declines and reductions in cognitive activity after retirement (23) or due to other aging-related changes in health and social relationships. The present findings not only contrast with the notion that there is little one can do to mitigate cognitive declines, but also shed light on previous inconsistent findings regarding the impact of educational attainment on aging-related cognitive decline (38). Some previous studies have found that individuals with lower educational attainment showed greater cognitive declines (18). The present findings suggest that not everyone with lower educational attainment experiences similar rates of cognitive declines, in part, because they may take active steps to reduce their declines by increasing their engagement in cognitive activities. Health care systems and health care professionals can play a key role in educating patients and their families about cognitive aging and the beneficial effects of frequent and increased engagement in cognitively stimulating activities in middle age and later life.

Although baseline cognitive activity was associated with changes in both EF and EM, the present findings show that beneficial effects of increased engagement in cognitive activities was only found for changes in EF. One possible explanation for this difference is that in the present study change in EF occurred earlier and was more salient compared to change in EM (4). Another possibility is that for EM the existing initial levels of cognitive activity were adequate or there may be other lifestyle factors that are more important for maintaining EM. To preserve EF seems to require going above and beyond previous levels of cognitive activity in order to afford protection for declines.

### Limitations and Future Directions

A first limitation of the longitudinal sample is the selective attrition of study participants. Typically, individuals who remain in longitudinal studies show positive bias compared to the attriters, which may restrict the observable range of variation in the longitudinal sample. Indeed, those who remained in the longitudinal MIDUS sample were better educated, healthier, had better cognitive functioning [cf. Hughes et al., (4)], and engaged in cognitive activities more often, which limits the generalizability of the findings as the extent of cognitive declines in the population may be underestimated.

A second limitation is the relatively broad assessment of cognitive activities. The cognitive activity measure used in this study focuses on the concurrent frequency of engaging in four types of cognitive activities, but does not provide any details on how demanding the specific tasks were for an individual. Alternative assessment approaches in future studies would be to measure cognitive activity with intensive time sampling by using daily activity diaries, direct observation of certain activities, or the use of observer reports. Future research is needed to test if different assessment approaches for cognitive activities reveal similar results and which cognitive activities are most useful for helping individuals attenuate aging-related cognitive declines. In addition, based on the present study, it is unclear how much individuals need to increase their frequency of engaging in cognitive activities to reap benefits, which needs to be tested systematically in the future.

Another consideration is the difficulty in separating cognitive activities from other forms of activities such as social activities. For example, social activities (e.g., playing card games) often involve cognitive engagement (38) and in turn, many cognitive activities (e.g., attending lectures) include social components. Recent research found that frequent contact with friends, but not family, was associated with greater concurrent engagement in physical and cognitive activities, which were both associated with better EM and EF (39). Future research should disentangle the nature of such experiences more thoroughly to test what specific aspects of activities can attenuate cognitive declines.

Finally, another limitation of the present study is that we only have two time points of repeated measures of cognitive activity and cognitive performance. The relationship between cognitive activities and cognitive performance represents a complex reciprocal process. Cognitive declines can also lead to curtailment of cognitive activities [e.g., (40)]. Future longitudinal studies with three or more occasions or intervention studies that target an increase in everyday cognitive activities are needed to more directly examine the directionality, causality, and reciprocity of this relationship. Although cognitive training studies have demonstrated that older adults can improve cognitive functioning when provided with intensive training in strategies that promote thinking and remembering [e.g., (41)], there is little transfer of function from specifically trained skills to new cognitive tasks, to reducing or reversing cognitive declines, or to maintaining any gains in cognitive functioning (38, 42). Additional research is needed to address the complexity of this reciprocal relationship and to test how an intellectually engaged lifestyle can best be promoted in everyday life.

## Conclusion

This research significantly extends prior cross-sectional research by testing whether increased engagement in cognitive activities is helpful for reducing cognitive declines and whether these effects varied by educational attainment. Adults who increased their cognitive activity showed less decline in EF across 9 years. However, further analysis showed differential results by educational level. For those with a college degree, cognitive declines did not significantly vary as a function of change in cognitive activity. In contrast, for those without a college degree, increasing cognitive activity was associated with less decline in EF. Thus, the results suggest that increased engagement in cognitive activity makes more of a difference for EF declines among those without a college degree. This emphasizes the importance of promoting and encouraging increased engagement in cognitive activity especially among those with lower educational attainment, who generally are at greater risk for cognitive impairment and less frequently engage in cognitive activities.

## Data Availability Statement

Publicly available datasets were analyzed in this study. This data can be found at: https://midus.colectica.org/; https://www.icpsr.umich.edu/web/ICPSR/search/studies?q=midus.

## Ethics Statement

The studies involving human participants were reviewed and approved by the Institutional Review Boards at the University of Wisconsin and Brandeis University. The patients/participants provided their written informed consent to participate in this study.

## Author Contributions

MS performed the data analysis, interpreted the findings, and drafted the manuscript. ML acquired the funding, interpreted the findings, and contributed to the writing of the manuscript. All authors substantially contributed to the research performed.

## Conflict of Interest

The authors declare that the research was conducted in the absence of any commercial or financial relationships that could be construed as a potential conflict of interest.

## Publisher's Note

All claims expressed in this article are solely those of the authors and do not necessarily represent those of their affiliated organizations, or those of the publisher, the editors and the reviewers. Any product that may be evaluated in this article, or claim that may be made by its manufacturer, is not guaranteed or endorsed by the publisher.
