# Supplementary material for: Increases in Cognitive Activity Reduce Aging-Related Declines in Executive Functioning
Source: Front Psychiatry. 2021 Jul 29;12:708974. doi: 10.3389/fpsyt.2021.708974 (PMC8358146; doi:10.3389/fpsyt.2021.708974)
Supplement: Supplementary file 1 [file Data_Sheet_1.docx]

Supplementary Material

# Supplementary Tables

Table S1. *Within-Occasion* *Correlations*

|  | 1 | 2 | 3 | 4 | 5 | 6 | 7 | 8 | 9 |
| --- | --- | --- | --- | --- | --- | --- | --- | --- | --- |
| (1) EM |  | .42** | .13** | .04 | .15** | -.39** | .14** | .17** | .18** |
| (2) EF | .38** |  | .14** | .07** | .32** | -.48** | .24** | .28** | .30** |
| (3) Cognitive activity | .16** | .20** |  | .44** | .22** | .13** | .12** | .00 | .09** |
| (4) Δ Cognitive activity | .06** | .03 | -.25** |  |  |  |  |  |  |
| (5) Education | .17** | .40** | .24** | .01 |  |  |  |  |  |
| (6) Age | -.26** | -.38** | .14** | -.02 | -.14** |  |  |  |  |
| (7) Self-rated health | .13** | .25** | .09** | .06** | .20** | -.08** |  |  |  |
| (8) Functional health | .12** | .26** | -.01 | .02 | .20** | -.27** | .51** |  |  |
| (9) Income | .13** | .29** | .03 | .07** | .30** | -.23** | .19** | .22** |  |
| (10) Physical activity | .12** | .22** | .08** | .03 | .19** | -.19** | .18** | .24** | .18** |
| (11) Sex | -.24* | .12** | -.15** | -.06* | .12** | .01 | .03 | .17** | .12** |
| (12) BMI | -.10** | -.10** | -.01 | -.01 | -.12** | .00 | -.28** | -.34** | -.07** |
| (13) Depression | .01 | .04 | -.02 | -.02 | -.03 | .10** | .19** | .16** | .06** |
| (14) Diabetes | -.12** | -.15** | -.01 | -.03 | -.05* | .15** | -.24** | -.19** | -.05* |
| (15) Head trauma | -.04 | -.00 | -.00 | .01 | -.03 | -.03 | -.07** | -.07** | -.04 |
| (16) Hypertension | -.11** | -.18** | .05* | -.05* | -.07** | .26** | -.24** | -.27** | -.11** |
| (17) Smoking | -.03 | -.10** | -.09** | -.02 | -.18** | .09** | -.15** | -.11** | -.12** |
| (18) Sleep | -.02 | .06* | -.03 | .01 | .09** | -.04 | .21** | .27** | .03 |
| (19) Vitamin C | .01 | -.06** | .06 | .00 | .04 | .14** | .03 | -.03 | -.03 |
| (20) Alcohol | -.03 | .02 | .00 | -.05* | .02 | .00 | -.03 | -.03 | -.03 |

*Note.* *N* = 2,130; * p < .05; ** p < .01; M2 (lower part); M3 (upper part). Cognitive activity change: 0 = decreased or maintained over time, 1 = increased over time; Education: 0 = without a college degree, 1 = with a college degree; Sex: 0 = female, 1 = male; Depression: 0 = no, 1 = yes; Diabetes: 0 = no, 1 = yes; Head trauma: 0 = no, 1 = yes; Hypertension: 0 = no, 1 = yes; Smoking: 0 = no, 1 = yes; Vitamin C: 0 = no, 1 = yes; Alcohol: 0 = no, 1 = yes.

Table S2. *Effects of Change in Cognitive Activity and Education on Change in EF without Covariates*

|  | Model 1 | | | | Model 2 | | | | Model 3 | | | | |
| --- | --- | --- | --- | --- | --- | --- | --- | --- | --- | --- | --- | --- | --- |
|  | *B* | *SE* | *p* | CI_95_ | *B* | *SE* | *p* | CI_95_ | *B* | *SE* | *p* | CI_95_ |  |
| Intercept | 0.38 | 0.02 | <0.001 | 0.34; 0.42 | 0.38 | 0.02 | <0.001 | 0.34; 0.42 | 0.14 | 0.04 | <0.001 | 0.07; 0.21 |  |
| Time | -0.26 | 0.01 | <0.001 | -0.28; -0.24 | -0.26 | 0.01 | <0.001 | -0.28; -0.24 | -0.30 | 0.02 | <0.001 | -0.33; -0.26 |  |
| Education | 0.29 | 0.02 | <0.001 | 0.25; 0.32 |  |  |  |  | 0.50 | 0.05 | <0.001 | 0.39; 0.60 |  |
| Δ Cognitive Activity |  |  |  |  | -0.05 | 0.03 | 0.034 | -0.10; -0.00 | -0.07 | 0.06 | 0.204 | -0.19; 0.04 |  |
| Education × Time | -0.01 | 0.01 | 0.574 | -0.03; 0.01 |  |  |  |  | 0.01 | 0.03 | 0.777 | -0.05; 0.06 |  |
| Δ Cognitive Activity × Time |  |  |  |  | 0.05 | 0.01 | <0.001 | 0.02; 0.07 | 0.11 | 0.03 | <0.001 | 0.05; 0.17 |  |
| Education ×  Δ Cognitive Activity × Time |  |  |  |  |  |  |  |  | -0.08 | 0.04 | 0.043 | -0.16; 0.01 |  |
| AIC | 6694.78 | | | | 7116.88 | | | | 6778.26 | | | | |
| BIC | 6732.92 | | | | 7155.02 | | | | 6841.82 | | | | |
| Log Likelihood | -3341.4 | | | | -3552.4 | | | | -3379.1 | | | | |
| *R^2^* Marginal | 0.156 | | | | 0.038 | | | | 0.159 | | | | |

*Notes.* Δ Cognitive activity: 0 = decreased and maintained cognitive activity over time, 1 = increased cognitive activity over time; Education: 0 = without a college degree, 1 = with a college degree; Fit indices of null model: AIC = 7126.51, BIC = 7151.94, Log likelihood = -3559.3; Cognitive activity: 0 = decreased and maintained cognitive activity over time, 1 = increased cognitive activity over time; Education: 0 = without a college degree, 1 = with a college degree.

Table S3. *Effects of Change in Cognitive Activity and Education on Change in EM without Covariates*

|  | Model 1 | | | | Model 2 | | | | Model 3 | | | |
| --- | --- | --- | --- | --- | --- | --- | --- | --- | --- | --- | --- | --- |
|  | *B* | *SE* | *p* | CI_95_ | *B* | *SE* | *p* | CI_95_ | *B* | *SE* | *p* | CI_95_ |
| Intercept | 0.08 | 0.05 | 0.101 | -0.02; 0.18 | 0.18 | 0.05 | <0.001 | 0.09; 0.27 | -0.02 | 0.06 | 0.731 | -0.15; 0.10 |
| Time | -0.13 | 0.03 | <0.001 | -0.18; -0.07 | -0.12 | 0.03 | <0.001 | -0.17; -0.07 | -0.10 | 0.04 | 0.009 | -0.17; -0.02 |
| Education | 0.32 | 0.07 | <0.001 | 0.18; 0.45 |  |  |  |  | 0.41 | 0.09 | <0.001 | 0.23; 0.59 |
| Δ Cognitive Activity |  |  |  |  | 0.14 | 0.07 | 0.049 | 0.00; 0.28 | 0.25 | 0.10 | 0.012 | 0.05; 0.45 |
| Education × Time | -0.01 | 0.04 | 0.832 | -0.09; 0.07 |  |  |  |  | -0.05 | 0.05 | 0.320 | -0.15; 0.05 |
| Δ Cognitive Activity × Time |  |  |  |  | -0.03 | 0.04 | 0.471 | -0.11; 0.04 | -0.08 | 0.06 | 0.151 | -0.20; 0.03 |
| Education ×  Δ Cognitive Activity × Time |  |  |  |  |  |  |  |  | 0.10 | 0.08 | 0.193 | -0.05; 0.26 |
| AIC | 10867.03 | | | | 10940.59 | | | | 10865.37 | | | |
| BIC | 10905.17 | | | | 10978.73 | | | | 10928.93 | | | |
| Log Likelihood | -5427.5 | | | | -5464.3 | | | | -5422.7 | | | |
| *R^2^* Marginal | 0.030 | | | | 0.007 | | | | 0.033 | | | |

*Notes.* Δ Cognitive activity: 0 = decreased and maintained cognitive activity over time, 1 = increased cognitive activity over time; Education: 0 = without a college degree, 1 = with a college degree; Fit indices of null model: AIC = 10943.91, BIC = 10969.34, Log likelihood = -5468.0; Cognitive activity: 0 = decreased and maintained cognitive activity over time, 1 = increased cognitive activity over time; Education: 0 = without a college degree, 1 = with a college degree.

|  | *B* | *SE* | *p* | CI_95_ |
| --- | --- | --- | --- | --- |
| Intercept | 3.03 | 0.03 | < 0.001 | 2.97; 3.10 |
| Time | 0.02 | 0.02 | 0.239 | -0.02; 0.06 |
| Education | 0.07 | 0.02 | 0.001 | 0.03; 0.11 |
| M2 Cognitive Activity | 1.16 | 0.03 | < 0.001 | 1.10; 1.22 |
| M2 Cognitive Activity × Time | -0.32 | 0.02 | <0.001 | -0.36; -0.28 |
| AIC | 4515.79 | | | |
| BIC | 4641.17 | | | |
| Log Likelihood | -2236.9 | | | |
| *R^2^* Marginal | 0.653 | | | |

Table S4. *Effects of M2 Cognitive Activity on Change in Cognitive Activity*

*Note.* Covariates are age, sex, income, physical activity, self-rated health, functional health, BMI, depression, diabetes, head trauma, hypertension, smoking, sleep problems, vitamin C, alcohol or drug problems.

Table S5. *Effects of M2 Cognitive Activity on Change in Executive Functioning (EF)*

|  | *B* | *SE* | *p* | CI_95_ |
| --- | --- | --- | --- | --- |
| Intercept | 0.26 | 0.03 | < 0.001 | 0.21; 0.31 |
| Time | -0.27 | 0.01 | < 0.001 | -0.30; -0.25 |
| Education | 0.27 | 0.03 | < 0.001 | 0.21; 0.33 |
| M2 Cognitive Activity | 0.17 | 0.02 | < 0.001 | 0.13; 0.22 |
| M2 Cognitive Activity × Time | -0.04 | 0.01 | 0.001 | -0.06; -0.02 |
| AIC | 4066.57 | | | |
| BIC | 4191.95 | | | |
| Log Likelihood | -2012.3 | | | |
| *R^2^* Marginal | 0.346 | | | |

*Note.* Education: 0 = without a college degree, 1 = with a college degree; Covariates are age, sex, income, physical activity, self-rated health, functional health, BMI, depression, diabetes, head trauma, hypertension, smoking, sleep problems, vitamin C, alcohol or drug problems.

|  | *B* | *SE* | *p* | CI_95_ |
| --- | --- | --- | --- | --- |
| Intercept | 0.17 | 0.04 | < 0.001 | 0.08; 0.26 |
| Time | -0.16 | 0.02 | < 0.001 | -0.20; -0.11 |
| Education | 0.13 | 0.04 | 0.001 | 0.05; 0.20 |
| M2 Cognitive Activity | 0.21 | 0.04 | < 0.001 | 0.13; 0.29 |
| M2 Cognitive Activity × Time | -0.05 | 0.02 | 0.038 | -0.10; -0.00 |
| AIC | 6846.53 | | | |
| BIC | 6971.91 | | | |
| Log Likelihood | -3402.3 | | | |
| *R^2^* Marginal | 0.215 | | | |

Table S6. *Effects of M2 Cognitive Activity on Change in Episodic Memory (EM)*

*Note.* Education: 0 = without a college degree, 1 = with a college degree; Covariates are age, sex, income, physical activity, self-rated health, functional health, BMI, depression, diabetes, head trauma, hypertension, smoking, sleep problems, vitamin C, alcohol or drug problems.

Table S7. *Effects of M2 Cognitive Activity and Increased Cognitive Activity on Change in Executive Functioning (EF)*

|  | *B* | *SE* | *p* | CI_95_ |
| --- | --- | --- | --- | --- |
| Intercept | 0.27 | 0.03 | < 0.001 | 0.20; 0.33 |
| Time | -0.27 | 0.02 | < 0.001 | -0.33; -0.27 |
| Education | 0.26 | 0.03 | < 0.001 | 0.21; 0.32 |
| M2 Cognitive Activity | 0.18 | 0.02 | < 0.001 | 0.13; 0.22 |
| M2 Cognitive Activity × Time | -0.03 | 0.01 | 0.012 | -0.06; -0.01 |
| Δ Cognitive activity | -0.01 | 0.05 | 0.842 | -0.10; 0.08 |
| Δ Cognitive activity x Time | 0.06 | 0.03 | 0.021 | 0.01; 0.11 |
| AIC | 4057.10 | | | |
| BIC | 4194.50 | | | |
| Log Likelihood | -2005.6 | | | |
| *R^2^* Marginal | 0.349 | | | |

*Note.* Δ Cognitive activity: 0 = decreased and maintained cognitive activity over time, 1 = increased cognitive activity over time; Education: 0 = without a college degree, 1 = with a college degree; Covariates are age, sex, income, physical activity, self-rated health, functional health, BMI, depression, diabetes, head trauma, hypertension, smoking, sleep problems, vitamin C, alcohol or drug problems.

Table S8. *Effects of M2 Cognitive Activity and Increased Cognitive Activity on Change in Episodic Memory (EM)*

|  | *B* | *SE* | *p* | CI_95_ |
| --- | --- | --- | --- | --- |
| Intercept | 0.09 | 0.06 | 0.121 | -0.02; 0.20 |
| Time | -0.12 | 0.03 | < 0.001 | -0.18; -0.05 |
| Education | 0.12 | 0.04 | 0.002 | 0.05; 0.20 |
| M2 Cognitive Activity | 0.24 | 0.04 | < 0.001 | 0.15; 0.32 |
| M2 Cognitive Activity × Time | -0.06 | 0.02 | 0.012 | -0.11; -0.01 |
| Δ Cognitive activity | 0.20 | 0.08 | 0.016 | 0.04; 0.37 |
| Δ Cognitive activity x Time | -0.10 | 0.05 | 0.051 | -0.20; 0.01 |
| AIC | 6844.60 | | | |
| BIC | 6981.90 | | | |
| Log Likelihood | -3399.3 | | | |
| *R^2^* Marginal | 0.216 | | | |

*Note.* Δ Cognitive activity: 0 = decreased and maintained cognitive activity over time, 1 = increased cognitive activity over time; Education: 0 = without a college degree, 1 = with a college degree; Covariates are age, sex, income, physical activity, self-rated health, functional health, BMI, depression, diabetes, head trauma, hypertension, smoking, sleep problems, vitamin C, alcohol or drug problems.

Table S9. *Effects of Change in Cognitive Activity on Change in EF Among Individuals with a College Degree*

|  | EF | | | |
| --- | --- | --- | --- | --- |
|  | *B* | *SE* | *p* | CI_95_ |
| Intercept | 0.55 (0.64) | 0.04 (0.04) | <0.001 (<0.001) | 0.46; 0.64 (0.56; 0.71) |
| Time | -0.30 (-0.28) | 0.02 (0.02) | <0.001 (<0.001) | -0.35; -0.25 (-0.33; -0.25) |
| Δ Cognitive Activity | 0.03 (-0.01) | 0.07 (0.06) | 0.659 (0.861) | -0.11; 0.17 (-0.12; 0.10) |
| Δ Cognitive Activity × Time | 0.02 (0.04) | 0.04 (0.03) | 0.554 (0.217) | -0.05; 0.09 (-0.02; 0.09) |
| AIC | 2115.76 (3470.47) | | | |
| BIC | 2226.42 (3504.58) | | | |
| Log Likelihood | -1036.9 (-1729.2) | | | |
| *R^2^* Marginal | 0.249 (0.045) | | | |

*Notes.* Δ Cognitive activity: 0 = decreased and maintained cognitive activity over time, 1 = increased cognitive activity over time;

Covariates = cognitive activity at M2, income, age, sex, frequency of physical activity, functional health, self-rated health, BMI, depression,

diabetes, head trauma, hypertension, smoking, sleep problems, vitamin C, alcohol or drug problems;

Without covariates in brackets; Fit indices of null model: AIC = 3469.53, BIC = 3492.27, Log likelihood = -1730.8.

Significant covariates are cognitive activity at M2 (*B* = 0.11, *SE* = 0.02, *p* < 0.001), income (*B* = 0.06, *SE* = 0.02, *p* = 0.002), age (*B* = -0.26, *SE* = 0.02, *p* < 0.001), self-rated health (*B* = 0.08, *SE* = 0.03, *p* = 0.003), and sex (*B* = -0.09, *SE* = 0.02, *p* < 0.001).

Table S10. *Effects of Change in Cognitive Activity on Change in EF Among Individuals Without a College Degree*

|  | EF | | | |
| --- | --- | --- | --- | --- |
|  | *B* | *SE* | *p* | CI_95_ |
| Intercept | 0.29 (0.14) | 0.04 (0.04) | <0.001 (<0.001) | 0.21; 0.37 (0.07; 0.21) |
| Time | -0.31 (-0.30) | 0.02 (0.02) | <0.001 (<0.001) | -0.35; -0.27 (-0.33; -0.25) |
| Δ Cognitive Activity | -0.09 (-0.07) | 0.06 (0.06) | 0.136 (0.202) | -0.22; 0.03 (-0.19; 0.04) |
| Δ Cognitive Activity × Time | 0.13 (0.11) | 0.03 (0.03) | <0.001 (<0.001) | 0.07; 0.20 (0.05; 0.17) |
| AIC | 1954.70 (3310.44) | | | |
| BIC | 2065.68 (3344.28) | | | |
| Log Likelihood | -956.4 (-1649.2) | | | |
| *R^2^* Marginal | 0.306 (0.0412) | | | |

*Notes.* Δ Cognitive activity: 0 = decreased and maintained cognitive activity over time, 1 = increased cognitive activity over time;

Covariates = cognitive activity at M2, income, age, sex, frequency of physical activity, functional health, self-rated health, BMI, depression,

diabetes, head trauma, hypertension, smoking, sleep problems, vitamin C, alcohol or drug problems;

Without covariates in brackets; Fit indices of null model: AIC = 3326.45, BIC = 3349.01, Log likelihood = -1659.2.

Significant covariates are cognitive activity at M2 (*B* = 0.14, *SE* = 0.02, *p* < 0.001), income (*B* = 0.09, *SE* = 0.03, *p* < 0.001), age (*B* = -0.24, *SE* = 0.02, *p* < 0.001), self-rated health (*B* = 0.09, *SE* = 0.02, *p* < 0.001), and sex (*B* = -0.06, *SE* = 0.02, *p* = 0.002).
